# Expand Your Horizon: Testing a Brief Writing Intervention Focused on Body Functionality Among Adolescent Girls With an Eating Disorder

**DOI:** 10.1002/eat.24444

**Published:** 2025-04-26

**Authors:** Klaske A. Glashouwer, Stella Weiland, Jessica M. Alleva

**Affiliations:** ^1^ Department of Clinical Psychology and Experimental Psychopathology University of Groningen Groningen the Netherlands; ^2^ Department of Eating Disorders Accare Child and Adolescent Psychiatry Groningen the Netherlands; ^3^ Department of Clinical Psychological Science Maastricht University Maastricht the Netherlands

**Keywords:** adolescents, body functionality, body image, eating disorders, intervention

## Abstract

**Objective:**

In the last decade, body image research has increasingly focused on the distinction between negative and positive body images as separate constructs. Functionality appreciation is a crucial component of positive body image and might be an important source for coping with body image problems. The intervention Expand Your Horizon (EYH; Alleva et al. 2015a) was designed to increase functionality appreciation and has demonstrated positive effects on body image across different adult populations. In the current research, we investigated the short‐term effects of a single‐session EYH in adolescent girls with an eating disorder.

**Method:**

Fifty‐eight adolescent girls with an eating disorder were randomly assigned to the EYH condition or an active control condition. Different aspects of positive and negative body images were assessed before and after the intervention.

**Results:**

Participants in the experimental condition showed higher scores than the control condition on positive body image after the writing exercise, but no effects on negative body image were found. Exploratory analyses showed that improvements in the positive body image remained stable 15–30 min after the writing exercise.

**Discussion:**

These outcomes suggest that writing about body functionality leads to direct increases in positive body image but not to decreases in negative body image. An important step for future studies would be to test the full three‐session version of EYH and investigate its effects over the longer term. This could help to determine the robustness of the present findings and the potential of EYH as a complementary intervention to existing treatments.

**Summary**

- We investigated whether adolescent girls with an eating disorder might profit from Expand Your Horizon (EYH), an intervention focusing on body functionality.
- We found that a single session of writing appreciatively about one's body functionality leads to improvements in positive body image but not to decreases in negative body image.
- An important next step will be to test the effects of several sessions of EYH and investigate its effects over the longer term.

Eating disorders are severe mental disorders characterized by a disturbance of food and body‐related attitudes and behaviors leading to impairments in emotional well‐being, physical health, and psychosocial functioning (van Hoeken and Hoek 2020; Schmidt et al. 2016). Lifetime prevalence of eating disorders lies between 1% and 2% in the general population depending on the specific disorder (Qian et al. 2013, 2022), with increased occurrence for young people (Silén and Keski‐Rahkonen 2022; Solmi et al. 2022). A key characteristic of anorexia nervosa (AN) and bulimia nervosa (BN) is a negative body image, which has been identified as one of the strongest predictors of the development and maintenance of these eating disorders (Beato‐Fernández et al. 2004; Cooley and Toray 2001; Dakanalis et al. 2017; Johnson and Wardle 2005; Stice 2002; Stice et al. 2017) and was found to be related both to treatment effectiveness and relapse (Carter et al. 2004; Glashouwer et al. 2019; Junne et al. 2019; Keel et al. 2005).

Body image is a complex construct that encompasses thoughts, behaviors, emotions, and evaluations related to one's body (Cash 2011). Over the past decade, research on body image has increasingly distinguished between negative and positive body image as separate constructs (Tylka and Wood‐Barcalow 2015a). Negative body image can manifest as a negative evaluation of, and preoccupation with, one's physical appearance (Cash 2011). In contrast, positive body image is defined by love, respect, and appreciation for one's body (Tylka and Wood‐Barcalow 2015a), and might be an important protective factor against the maintenance and relapse of eating disorders (Linardon 2021; Messer et al. 2022). Functionality appreciation is a crucial component of positive body image, and is defined as “appreciating, respecting, and honouring the body for what it is capable of doing, extending beyond mere awareness of body functionality” (Alleva et al. 2017, 29). Body functionality encompasses everything the body can do, including bodily senses, physical capacities, internal processes, creative activities, communication with others, and self‐care/daily routines (Alleva and Tylka 2021). Functionality appreciation likely contributes to lower levels of eating disorder symptoms, higher levels of adaptive eating (e.g., intuitive eating), and better mental health and well‐being (Linardon et al. 2023). Individuals with AN and BN typically evaluate their self‐worth based predominantly on their weight, shape, eating, and their control (Fairburn et al. 2003), and increasing the awareness and appreciation of body functionality could help them become aware that “their body is more than its appearance” and broaden the self‐evaluation to other domains. In addition, shifting the focus from appearance to valuable functions of the body could counter the societal tendency to objectify the female body (Fredrickson and Roberts 1997).

Expand Your Horizon (EYH) was developed to increase functionality appreciation and consists of 3 sessions (15 min each) of writing about what one's body can do and why those body functions are personally meaningful (Alleva et al. 2015a). Each session concerns two different domains of body functionality (e.g., bodily senses and creative activities). EYH was originally investigated in adult women with a negative body image (Alleva et al. 2015a). Compared to the active control condition, participants in the EYH condition reported higher appearance satisfaction, functionality appreciation, and body appreciation, along with lower levels of self‐objectification immediately after the intervention and 1 week later. In addition, EYH has also demonstrated positive effects on body image in women with rheumatoid arthritis (Alleva et al. 2018), adults with visible differences (Guest et al. 2024), women high in weight bias internalization (Davies et al. 2022), and women who had undergone bariatric surgery (Alleva et al. 2023a, 2023b). Further, a single‐session version of EYH (covering all domains of body functionality in a 15–30‐min writing exercise) has also demonstrated positive effects on functionality appreciation and body appreciation in adult women (e.g., Alleva et al. 2016a, 2016b). The single‐session EYH also improved body image in mothers of young children (Granfield et al. 2023).

In summary, several studies have demonstrated positive effects on body image both for the original and shortened version of EYH across different populations. In fact, a systematic review showed that EYH is currently the most effective intervention for improving positive body image in adults (Guest et al. 2019). However, EYH has not yet been investigated in clinical groups of individuals with an eating disorder nor in young individuals (Guest et al. 2022), despite the fact that adolescence is a critical period for the development of body image problems (Möllmann et al. 2024) and that eating disorders are highly prevalent in this age group with a peak onset at 15.5 years (e.g., Solmi et al. 2022). Given the persistence of negative body image within eating disorders and the need for more effective treatments, it is important to investigate the effects of EYH in young individuals with an eating disorder. While body dissatisfaction affects both genders, women predominantly wish to be thinner, whereas men equally desire weight loss or muscle gain (Cash 2000). Additionally, eating disorders are 4–10 times more prevalent in women (Smink et al. 2012; Qian et al. 2013). Although boys could theoretically also benefit from the intervention, we chose to focus on adolescent girls to enhance feasibility, ensuring an adequate sample size and facilitating interpretation of the outcome measures within a more homogeneous group in terms of body image concerns.

Previous studies have raised concerns that this intervention could unintentionally draw attention to negative body aspects. To minimize this risk, the current research was set up as a proof‐of‐principle study to investigate the short‐term effects of the single‐session EYH in 58 adolescent girls with an eating disorder (i.e., AN, BN, or an eating disorder not otherwise specified). Participants were randomly assigned to the EYH condition or to an active control condition. We hypothesized that compared to the control condition, individuals in the experimental condition would show (i) higher scores in *positive body image* (i.e., functionality appreciation and body appreciation) and (ii) lower scores in *negative body image* (i.e., appearance dissatisfaction and overevaluation of shape and weight). These primary outcome measures were selected because they represent key components of positive and negative body image (Glashouwer et al. 2019; Tylka and Wood‐Barcalow 2015a; Swami et al. 2020). Additionally, as a secondary outcome, we examined whether EYH leads to (iii) lower scores in *dietary restriction*, an important core symptom of both AN and BN. This was included to explore whether EYH also could lead to improvements in eating disorder behaviors. Further, we explored whether any effects on body image remained 15 and 30 min after the writing exercise. Last, using a cross‐over design in which individuals in the control condition received EYH after completing the control exercise, we explored whether any potential positive effects in the experimental condition would be replicated.

## 1 Methods

### 1.1 Participants

Fifty‐eight adolescent girls with an eating disorder (*M*_*age*_ = 16.76, SD = 1.81, Range = 14.24 to 23.96 years) were recruited through the Department of Eating Disorders of Accare, a mental health care facility for children and adolescents in the Netherlands. We included a wide age range for feasibility, aiming to include all eligible participants from our healthcare institute. Additionally, we had no theoretical reasons to assume the intervention would be more or less effective in younger vs. older adolescents. Inclusion criteria were (i) female sex, (ii) 14–23 years old, (iii) currently receiving treatment, (iv) being fluent in Dutch, and (v) being formally diagnosed with AN, BN, or an eating disorder not otherwise specified by a health care professional of Accare based on the DSM‐5 criteria and with the use of the Child Eating Disorder Examination (ChEDE; Bryant‐Waugh et al. 1996; Decaluwé and Braet 1999). Participants were undergoing treatment for AN of the restrictive type (*n* = 18), AN of the purging type (*n* = 3), atypical AN (*n* = 4), BN (*n* = 6), or another specified eating disorder with features of AN or BN (*n* = 24); the specific classification of three participants was unknown^1^. Participants were randomized to the experimental (*n* = 31) or control (*n* = 27) condition. The study was approved by the Medical Ethical Committee of the University Medical Center Groningen (NL64270.042.18) and was preregistered in the Dutch Trial Register (NTR7704). Data were collected between January 2019 and February 2024.

### 1.2 Measurements

#### 1.2.1 Primary Outcome Measures

##### 1.2.1.1 Functionality Appreciation

State functionality appreciation was measured using two visual analogue scale (VAS) items (cf. Alleva et al. 2016a, 2016b). Participants indicated their current level of satisfaction and dissatisfaction (reverse‐coded) with their “body functionality (i.e., everything your body can do)” by sliding a bar on the computer screen (0 = *none* to 100 = *extreme*). Participants' responses to the two VAS items were averaged; higher scores indicate higher functionality appreciation. The internal consistency for these items was poor with 0.51 (pretest) and 0.29 (post‐test), and therefore the items were explored separately in more detail (see Results).

##### 1.2.1.2 Body Appreciation

State body appreciation was assessed with two items adapted from the Body Appreciation Scale‐2 (cf. Alleva et al. 2016a, 2016b; Tylka and Wood‐Barcalow 2015b). Participants indicated whether they currently appreciate or have little appreciation (reverse‐scored) for “the different and unique characteristics of their body.” The items were rated and scored in the same manner as the aforementioned items; higher scores represent higher body appreciation. The internal consistency for these items was rather low, with 0.59 (pretest) and 0.68 (post‐test). Again, the items were also explored separately in detail.

##### 1.2.1.3 Appearance Dissatisfaction

State appearance dissatisfaction was measured using two VAS items (cf. Alleva et al. 2016a, 2016b; Birkeland et al. 2005). Participants indicated their current level of satisfaction (reverse‐scored) and dissatisfaction with their appearance. The items were rated and scored in the same manner as the aforementioned items; higher scores represent higher appearance dissatisfaction. The internal consistency was good for the pretest with 0.84 and acceptable for the post‐test with 0.73.

##### 1.2.1.4 Overevaluation of Weight and Shape

State overevaluation of weight and shape was assessed with two items adapted from the Eating Disorder Examination Questionnaire 6.0 (EDE‐Q; Fairburn and Beglin 2008). Participants indicated to what extent they agree at this moment with the statements “my weight influences how I think about myself” and “my shape influences how I think about myself,” rated from 0 (*Not at all*) to 6 (*Markedly*). Item scores were averaged; higher scores indicate higher overevaluation of weight and shape. Internal consistency was good with 0.84 (pretest) and 0.84 (post‐test).

Since overevaluation of weight and shape might be a complicated concept for adolescents, we included an additional assessment adapted from the ChEDE. Namely, participants were asked to think about things that are important for the way they think about themselves at this moment, and to write these on five or six post‐it notes. Then, participants ranked the notes in order of importance. The researcher scored the aspects of shape and weight according to the ChEDE guidelines, ranging from 0 (*not important at all*) to 6 (*exceptionally important*), to indicate how much value was placed on them. For example, when a participant ranked shape as the most important, it received a score of 6, the second most important received a score of 5, and so on. Participants' rankings for both aspects were averaged; higher scores indicate higher overevaluation of weight and shape.

#### 1.2.2 Secondary Outcome Measure

State intention for food restriction was assessed with two items adapted from the Restraint Scale of the EDE‐Q (Fairburn and Beglin 2008). Participants indicated on two VAS scales to what extent at this moment they “are planning to limit the amount of food they eat” and “are planning to limit the intake of high‐caloric foods they like.” The items were rated and scored in the same manner as the aforementioned VAS items; higher scores represent higher intention for food restriction. The internal consistency for these items was acceptable for the pretest with 0.77 and good for the post‐test with 0.82.

#### 1.2.3 Validation of the Outcome Measures

The measures for *functionality appreciation* and *appearance dissatisfaction* were identical to those used in a study on Dutch undergraduate students with a mean age of 20.6 years (Alleva et al. 2016a, 2016b), where internal consistencies were excellent. The two items for *body appreciation* were adapted from the Body Appreciation Scale‐2, which has been validated in Dutch undergraduate students with a mean age of 21.3 years (Alleva et al. 2016a, 2016b). *Overevaluation of weight and shape*, as well as *intention for food restriction*, were derived from the Eating Disorder Examination Questionnaire, a widely used measure across different populations, including Dutch adolescents with eating disorders. While the measures have not been specifically validated in our exact study population, they have been used in other groups, demonstrating good internal consistency.

#### 1.2.4 Descriptives

##### 1.2.4.1 Demographics

Participants reported their age, sex, and educational level. Diagnostic information (i.e., type of diagnosis and subtype) was derived from their medical folder. As body mass index (BMI; weight/height^2^) in children and adolescents changes with age, we calculated adjusted BMI scores ([actual BMI/The 50th percentile of BMI for age and gender] x100) (Le Grange et al. 2012). We obtained the 50th percentile of BMI for age and gender from the Netherlands Organization for Applied Scientific Research (TNO 2010).

##### 1.2.4.2 Eating Disorder Symptoms

The severity of eating disorder symptoms was indexed by the EDE‐Q 6.0 (Fairburn and Beglin 2008), which provides a global measure of the severity of eating disorder pathology over the last 28 days. Items are scored from 0 (*No days/Not at all*) to 6 (*Every day/Markedly*). Item scores are averaged, with higher scores indicating more eating disorder symptoms (cf. Aardoom et al. 2012). The internal consistency was 0.94.

### 1.3 Intervention

#### 1.3.1 Expand Your Horizon

We used a one‐session version of EYH (cf. Alleva et al. 2016a, 2016b). We slightly adjusted the language to make it more understandable and relevant to adolescents (e.g., using “girls” instead of “women”) and offered a more extended explanation of the concept of body functionality at the beginning to ensure that the participants fully understood it. The writing exercise was administered on a computer using Qualtrics (Qualtrics 2018). At the start, participants received a brief description of body functionality and a list of example body functions. Subsequently, participants were instructed to write in detail about everything their body can do and why those functions are personally meaningful. If they needed inspiration during the exercise, they could look at the list with examples of body functions. After the general introduction about body functionality, the writing exercise consisted of three subparts, each focused on two domains of body functions: (1) bodily senses and physical capacities, (2) internal processes and creativity, and (3) self‐care/daily routines and communication. The instructions were for example “What can you do with your body in terms of (1) senses and sensations and (2) physical activity and movement? What does this mean to you?.” After finishing the exercise, participants were asked to think about and write down two actions that could help them to maintain their focus on the functionality of their bodies.

#### 1.3.2 Active Control

Participants in the active control condition received an explanation about information processing and were asked to reflect on both the conscious and subconscious information they encounter throughout the day. After a general introduction, they were asked to write about detail perception during a familiar route (e.g., home to school) and were given a list of example details (cf. Alleva et al. 2016a, 2016b) in the categories: (1) nature and time, (2) buildings, and (3) transportation and traffic. After completion, participants noted down two actions they could take in the near future to engage more with detail perception.

Both groups were given 15 min for their writing and were encouraged to use the full time and not to worry about spelling and grammar. A review of the writing exercise responses suggested that participants indeed engaged with the task and followed the instructions for their respective condition. A qualitative analysis of these writings is reported in a separate paper.

### 1.4 Procedure and Design

**FIGURE 1:**
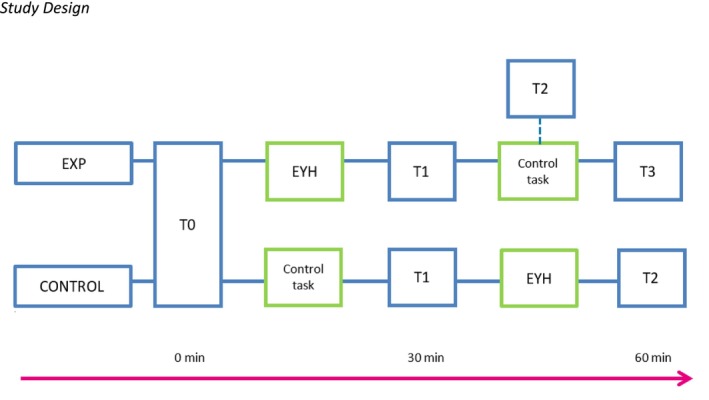
Study design. CONTROL, control condition; EYH, Expand Your Horizon; EXP, experimental condition.

Individuals who met the inclusion criteria were informed about the study by their practitioner. Participants (and, if younger than 16 years, their parents or a guardian with parental authority) actively gave informed consent before the start of the study. Participants were informed about the procedure but not the specific conditions or hypotheses. They were told that the study aimed to explore the impact of writing on how you feel about yourself and to examine different writing styles, as writing can help express thoughts and feelings. The study was performed at the healthcare institute. After the baseline assessment, participants were randomized to a condition. We used block randomization with blocks of 12 participants to ensure a balanced number of participants per condition. The study used a cross‐over design (see Figure 1): After completing the first condition (i.e., the EYH or control exercise), the participants also received the other condition, so that eventually all participants completed the EYH exercise. The descriptive measures were completed at baseline (T0)^2^. At baseline (T0), post‐test intervention 1 (T1), halfway intervention 2 (experimental condition: T2), post‐test intervention 2 (experimental condition: T3; control condition: T2), participants completed the primary and secondary outcome measures with the exception of the ranking task (overevaluation of weight and shape), which was completed at post‐test (T1) only. Participants received an €8 voucher for their participation.

### 1.5 Statistical Analysis

The data are available upon request from the first author and will be made publicly available via https://dans.knaw.nl/after publication.

#### 1.5.1 Power Calculation

When performing a power calculation using G‐power 3.1 for the ANCOVA analyses for the primary outcome measures with a medium effect size (*η* ^2^ = 0.08 cf. Alleva et al. 2015a), an alpha of 0.05 and power of 0.80, 93 participants would have been required. Unfortunately, we did not manage to reach the total sample.

#### 1.5.2 Hypothesis Testing

Four separate ANCOVAs were conducted with pretest scores as covariate (T0), post‐test as dependent variable (T1), and Condition (experimental vs. control) as independent variable. Appearance dissatisfaction slightly deviated from normality, but we expected the ANCOVA to be robust for these small deviations considering the group sizes. For functionality appreciation and appearance dissatisfaction, the assumption of equal regression slopes was violated. In these cases, we added an interaction term to test if the interaction between pretest and Condition was significant (cf. Cohen et al. 2003). This was not the case, and therefore we left the interaction terms out of the final analyses. Differences on the ranking task (overevaluation of weight and shape) were tested with a Mann–Whitney U test, since the data were not continuous. A Bonferroni–Holm correction was applied to control for increased familywise error rate: For the primary outcomes, the smallest *p*‐value was tested against an alpha of 0.01, following against 0.0125, 0.0167, 0.025, and 0.05. The difference on the secondary outcome measure was tested with an independent samples t‐test. In the case of outliers according to the Tukey fence method (Tukey 1977), the analyses were repeated without the outliers. Removing outliers did not lead to any changes in significance testing, and the presented results are therefore including outliers.

#### 1.5.3 Exploratory Analyses

We explored whether any effects on body image remained 15 and 30 min after the writing exercise in the experimental condition by performing a RM‐ANOVA with Time (mean scores at T1, T2, and T3) as within‐subject variable. Furthermore, we used paired samples t‐tests to explore whether any potential positive effects in the experimental condition would be replicated in individuals in the control condition who later received EYH.

#### 1.5.4 Bayesian Analyses

As we failed to reach the required sample size, we complemented the statistical analyses with the Bayesian approach to quantify the evidence regarding the null hypothesis using the free software JASP with default priors (JASP Team 2024). We reported BF_01_ for non‐significant tests which quantifies the evidence for the null hypothesis (i.e., condition has no effect on the outcome measure over and above T1 scores of the dependent variable) over the alternative hypothesis (i.e., condition has an effect on the outcome measure over and above T1 scores of the dependent variable). A Bayes factor of 1 is considered no evidence, between 1 and 3 *anecdotal*, between 3 and 10 *moderate*, between 10 and 30 *strong*, between 30 and 100 *very strong*, and more than 100 *extreme* evidence that the data are more likely under the null hypothesis (Wagenmakers et al. 2017).

#### 1.5.5 Missing Data

There were no missing data, as the study was conducted in a single session. In addition, there were no missing responses on items in the survey. This was ensured by the way the online questionnaire tool was set up. Specifically, participants could not proceed to the next item unless all previous items had been answered, thus preventing any missing responses.

## 2 Results

### 2.1 Primary Outcome Measures

#### 2.1.1 Functionality Appreciation

Participants in the experimental condition reported higher functionality appreciation than participants in the control condition at post‐test (see Table 1 for the descriptives for all outcome measures). Yet, this difference did not reach the adjusted significance level, which was set at *p* < 0.0125 (*F*(1,55) = 5.42, *p* = 0.024, *η* ^*2*^ *p* = 0.09, BF_01_ = 0.47). The effect size indicates a medium to large effect, and the Bayes factor suggests anecdotal evidence that the data are more likely under the alternative hypothesis (i.e., that the conditions *do* differ).

**TABLE 1:** Means and standard deviations per condition.

	Experimental condition (*n* = 31)				Control condition (*n* = 27)		
	M (SD)				M (SD)		
Descriptives							
Age	16.71 (1.99)				16.83 (1.62)		
Adjusted BMI	95.87 (12.36)^a^				94.80 (15.49)		
EDE‐Q	3.44 (1.51)^b^				3.59 (1.09)		
Diagnoses (*n*, %)							
AN restrictive type	10 (32.3%)				8 (29.6%)		
AN purging type	2 (6.5%)				1 (3.7%)		
Atypical AN	4 (12.9%)				0		
BN	4 (12.9%)				2 (7.4%)		
Other specified eating disorders	10 (32.3%)				14 (51.9%)		

Primary outcome measures	Pretest (T0)	Post‐test (T1)	Post‐test (T2)	Post‐test (T3)	Pretest (T0)	Post‐test (T1)	Post‐test (T2)
Functionality appreciation	49.34 (20.42)	65.63 (16.69)	67.53 (17.13)	65.94 (17.49)	56.39 (18.81)	59.06 (16.50)	65.20 (19.35)
Body appreciation	44.32 (20.46)	58.02 (19.42)	62.18 (18.37)	61.02 (20.56)	34.78 (21.05)	41.80 (20.56)	56.0 (19.70)
Appearance dissatisfaction	69.34 (25.04)	65.13 (24.51)	68.84 (24.0)	66.61 (23.57)	75.69 (14.92)	71.09 (20.43)	68.93 (18.4)
Overevaluation of shape and weight	5.45 (1.59)	5.18 (1.74)	5.13 (1.87)	5.19 (1.89)	5.91 (1.03)	5.46 (1.29)	5.28 (1.36)
Ranking of shape and weight (*Median, IQR*)	5.0 (3.25–5.50)				4.0 (3.00–5.50)		

*Note*: Most participants followed higher secondary education (*n* = 32), followed by middle secondary education (*n* = 12), pre‐vocational secondary education (*n* = 6), higher vocational education (*n* = 3), and university (*n* = 2). Three participants did not provide their education level.

^a^ Adjusted body mass index scores ([actual BMI/The 50th percentile of BMI for age and gender] x 100). One participant did not report her weight.

^b^ EDE‐Q, mean score on the Eating Disorder Examination Questionnaire.

#### 2.1.2 Body Appreciation

Participants in the experimental condition reported higher body appreciation than the control condition at post‐test (Table 1). Yet, this difference did not reach the significance level, which was *p* < 0.01 (*F*(1,55) = 6.03, *p* = 0.017, *η* ^*2*^ *p* = 0.11, BF_01_ = 0.28). The effect size indicates a medium to large effect. The Bayes factor suggests moderate evidence that the data are more likely under the alternative hypothesis.

#### 2.1.3 Appearance Dissatisfaction

At post‐test, participants in the experimental condition reported lower appearance dissatisfaction than the control condition (Table 1). Again, this difference did not reach the significance level, which was *p* < 0.05 (*F*(1,55) = 0.07, *p* = 0.80, *η* ^*2*^ *p* = 0.001, BF_01_ = 3.61). The effect size indicates a small effect, and the Bayes factor suggests moderate evidence that the data are more likely under the null hypothesis.

#### 2.1.4 Overevaluation of Shape and Weight

At post‐test, participants in the control condition reported higher overevaluation of shape and weight (Table 1), but this difference did not reach the significance level of *p* < 0.0167 (*F*(1,55) = 0.96, *p* = 0.33, *η* ^*2*^ *p* = 0.02, BF_01_ = 2.52). The effect size indicates a small effect, and the Bayes factor suggests anecdotal evidence that the data are more likely under the null hypothesis. In addition, the conditions did not differ on the ranking task (*U* = 357.50, *p* = 0.45, BF_01_ = 3.42).

### 2.2 Secondary Outcome Measure

There were no statistically significant differences between the experimental (*M =* 56.82, SD = 31.43) and the control condition (*M* = 54.39, SD = 27.92) on the intention to restrict food intake (*t*(56) = −0.31, *p* = 0.76, *d* = −0.082).

### 2.3 Post Hoc Analyses

Considering the low internal consistency of the functionality appreciation and body appreciation items, we inspected the items separately and noticed that some individuals answered very inconsistently. Therefore, we explored post hoc if these inconsistent answers could have influenced the results. Namely, we excluded participants who either scored very high (= > 65) or very low (= < 35) on both items. After exclusion of 12 participants, the internal consistency for functionality appreciation was 0.82 (pretest) and 0.79 (post‐test). The ANCOVA showed a statistically significant large effect of Condition on functionality appreciation (*F*(1,43) = 11.95, *p* = 0.001, *η* ^*2*^ *p* = 0.22, BF_10_ = 19.06). Participants in the experimental condition showed higher functionality appreciation (*M* = 67.76, SD = 17.66) than participants in the control condition (*M* = 59.50, SD = 17.17) at post‐test. Further, after excluding 13 participants, the internal consistency for body appreciation increased to 0.87 (pretest) and 0.91 (post‐test). The outcomes of the ANCOVA were comparable to the uncorrected analysis (*F*(1,42) = 5.69, *p* = 0.022, *η* ^*2*^ *p* = 0.12, BF_01_ = 0.32; experimental condition: *M* = 60.45, SD = 21.12; control condition: *M* = 40.83, SD = 21.04 at post‐test).

### 2.4 Exploratory Results

#### 2.4.1 Stability of Effect Over Time

The RM‐ANOVA showed no statistically significant effect of Time on functionality appreciation (*F*(2,60) = 0.20, *p* = 0.82; Mean T1 = 65.63, Mean T2 = 67.53, Mean T3 = 65.94)^3^ or on body appreciation (*F*(2,60) = 1.60, *p* = 0.21; Mean T1 = 58.02, Mean T2 = 62.18, Mean T3 = 61.02) after the writing exercise, supporting that potential improvements in functionality appreciation and body appreciation were sustained.

#### 2.4.2 Effects in Control Condition

There was no significant difference in functionality appreciation from before (*M* = 59.06, SD = 16.50) to after (*M* = 65.20, SD = 19.35) the EYH exercise for individuals in the control condition (*t*(26) = 1.64, *p* = 0.11, *d* = 0.32)^4^, but there was a significant large increase in body appreciation scores (*t*(26) =4.41, *p* = < 0.001; *d* = 0.85; before: *M* = 41.80, SD = 20.56; after: *M* = 56.00, SD = 19.72).

## 3 Discussion

We examined whether a single‐session version of EYH (Alleva et al. 2015, 2016a, 2016b) could improve body image among adolescent girls with an eating disorder. As expected, individuals in the experimental condition tended to score higher than the control condition on positive body image (i.e., functionality appreciation and body appreciation) after the writing exercise. However, we did not find an effect on negative body image (i.e., appearance dissatisfaction and overevaluation of shape and weight). In addition, exploratory analyses showed that the improvements in positive body image remained stable 15 and 30 min after the writing exercise. Finally, we explored whether the positive effects in the experimental condition would be replicated among individuals in the control condition who received EYH after completing the control exercise. Indeed, findings were in the same direction as in the experimental condition, although only the effect on body appreciation was significant.

These findings are in line with prior findings of the original three‐session version of EYH in adult women with a negative body image (Alleva et al. 2015a) and women with rheumatoid arthritis (Alleva et al. 2018). Also, a study investigating three sessions of EYH in adults with visible differences found similar improvements in functionality appreciation, but not in body appreciation (Guest et al. 2024); and a study in women high in weight bias internalization found improvements in functionality appreciation (body appreciation was not assessed; Davies et al. 2022). In addition, the present findings are in line with two prior studies investigating the one‐session version of EYH in adult women (Alleva et al. 2016a, 2016b) and mothers of young children (Granfield et al. 2023). Although the differences between conditions formally did not reach the adjusted significance levels, the magnitude of the effect sizes in the present study was comparable to effect sizes in prior studies using the three‐session EYH (Alleva et al. 2015a; Guest et al. 2024; Davies et al. 2022) and seemed larger than in prior studies using the single‐session EYH (Alleva et al. 2016a, 2016b; Granfield et al. 2023). This may be related to the greater severity of body image concerns in the present sample, which may have allowed for more room for improvement (cf. Alleva et al. 2015a). Together with the outcomes of the (post hoc) Bayesian statistics, these findings support the conclusion that one session of EYH shows positive short‐term effects on positive body image among adolescent girls with an eating disorder.

We did not find support that one session of EYH leads to reductions in negative body image. This is in contrast with two prior studies that found reductions in appearance dissatisfaction after three sessions of EYH (Alleva et al. 2015a, 2018). However, three sessions of EYH in women who have undergone bariatric surgery also did not lead to an effect on appearance satisfaction (Alleva et al. 2023a, 2023b) and one session of EYH did not change appearance satisfaction in adult women (Alleva et al. 2016a, 2016b). No studies so far have examined the effect of EYH on overevaluation of weight and shape. The current findings suggest that adolescent girls with an eating disorder can increase the appreciation toward their own body (functionality), and that these aspects of positive body image can exist alongside appearance dissatisfaction (Tylka and Wood‐Barcalow 2015a). Additional writing sessions may be necessary to sufficiently boost positive body image, after which the effects could “spill over” to negative body image aspects. Prior studies indeed found an effect on appearance satisfaction after three sessions of EYH (Alleva et al. 2015a, 2018), although still the effects on appearance satisfaction seemed smaller than on functionality appreciation (Alleva et al. 2015a). Theory and empirical findings suggest that in individuals with eating disorders, overevaluation of weight and shape is rather stable, hard to change in treatment, and closely tied to self‐esteem (e.g., Fairburn et al. 2003)—thus, change in functionality appreciation may need to be stronger, or be experienced for longer, to affect the overvaluation of weight and shape. The same may apply to the intention to restrict food intake, for which no effect was found either. It is possible that multiple sessions are required before improvements in body image translate into changes in (intention to engage in) eating disorder behaviors, such as dieting.

Collectively, these findings suggest that increasing the awareness and appreciation of body functionality may be a promising approach in the treatment of eating disorders. Potentially, functionality appreciation might help to foster motivation to take care of one's body and to let go of eating disorder behaviors (cf. Linardon et al. 2023). In support, a qualitative study among women who perceived their body to differ from societal norms—many of whom had an eating disorder in the past—described functionality appreciation as a key process in their “journey” from a predominantly negative toward a predominantly positive body image (Alleva et al. 2023a, 2023b). Part of this process involved a growing conviction that one's body is “more than one's appearance,” and is connected to their inherent values and broader purpose in life. An important direction for future research could be to investigate whether EYH could be more impactful when body functionality is explicitly linked to one's most meaningful goals and values. That way, functionality appreciation could help to put the body in a totally different perspective from “being dissatisfied with its weight and shape” to “being a crucial part of experiencing a meaningful life” (e.g., van Doornik et al. 2024; Glashouwer and de Jong 2024).

One limitation of this study was the low internal consistency of the functionality appreciation and body appreciation assessments. We noticed that some individuals showed very inconsistent scores, suggesting that some participants might have mistakenly interpreted both items as the same question. After repeating the analyses excluding these individuals, we found that, if anything, the effects of EYH increased. In future studies, it will be important to remind participants to carefully read each statement before responding, or to consider alternative assessments (see, e.g., Alleva et al. 2024a, 2024b). In particular, utilizing formally validated sets of items from larger psychometric studies could help ensure reliability and validity. A second limitation was the small sample size. To facilitate the interpretation and increase confidence in the present findings, we included Bayesian statistics. Furthermore, we examined the effect sizes and compared these with effect sizes in prior studies. Nevertheless, future studies are needed to be able to corroborate the robustness of these outcomes. Finally, our confidence in this intervention approach was based on prior research using the single‐session variant, including analysis of participant responses indicating positive body image outcomes. In earlier studies, participants also provided input on aspects such as exercise length and number, particularly in relation to the three‐session version. However, we acknowledge the limitation that this feedback was not obtained from adolescents with eating disorders, who may have different perspectives and needs.

## 4 Summary

We investigated whether adolescent girls with an eating disorder might profit from an intervention emphasizing functionality appreciation. As a first step, we studied the short‐term effects of a single EYH writing session (Alleva et al. 2016a, 2016b). The findings suggest that writing appreciatively about one's body functionality leads to increases in positive body image, but not to decreases in negative body image. An important step for future studies is to test the full three‐session version of EYH and investigate its longer‐term effects. This could help to determine the robustness of the present findings and investigate the potential of EYH as a complement to existing body image/eating disorder treatments. Moreover, it might be fruitful to have individuals more explicitly link their body functions to their core goals and intrinsic values to further facilitate functionality appreciation as a potential turning point in the journey from negative toward positive body image.

## Author Contributions

**Klaske A. Glashouwer:** conceptualization, investigation, methodology, supervision, writing – original draft. **Stella Weiland:** data curation, formal analysis, writing – review and editing. **Jessica M. Alleva:** conceptualization, methodology, writing – review and editing.

## Disclosure

No potential competing interest was reported by the authors. Part of this research was supported by a Veni grant [451–15‐026] awarded by the Dutch Research council (NWO). Preparation of this article by the second author was supported by an SSH Open Competition M grant [406.22.GO.006] awarded by the Dutch Research council (NWO).

## Conflicts of Interest

The authors declare no Conflicts of Interest.

## Data Availability

The data are available upon request from the first author and will be made publicly available via https://dans.knaw.nl/ after publication.

## Endnotes

^1^
Before recruitment, therapists anonymously confirmed participants' eating disorder diagnoses. The missing classifications arose later when, despite consent, some patient records lacked accurate data for formal verification.

^2^
In addition, the following questionnaires were administered at baseline: Body Appreciation Scale‐2 (Tylka and Wood‐Barcalow 2015b); Functionality Appreciation Scale (Alleva et al. 2017); Single Item Self‐Esteem Scale (Robins et al. 2001). Outcomes will be reported in a separate paper.

^3^
Normality was slightly violated, but we deemed the RM‐ANOVA to be robust for this violation.

^4^
Similar outcomes were found after post hoc excluding four inconsistently scoring participants (*t*(22) = 1.49, *p* = 0.15, *d* = 0.31).
